# Early Mortality Predictors in İnfective Endocarditis Patients: A Single-Center Surgical Experience

**DOI:** 10.21470/1678-9741-2021-0621

**Published:** 2022

**Authors:** Çiğdem Tel Üstünışık, Zihni Mert Duman, Barış Timur, Timuçin Aksu, Taner İyigün, Safa Göde, Muhammed Bayram, Vedat Erentuğ

**Affiliations:** 1 Department of Cardiovascular Surgery, Istanbul University Cerrahpasa Faculty of Medicine, Istanbul, Turkey.; 2 Department of Cardiovascular Surgery, Cizre State Hospital, Şırnak, Turkey.; 3 Department of Cardiovascular Surgery, Istanbul Dr. Siyami Ersek Thoracic and Cardiovascular Surgery Training and Research Hospital, Istanbul, Turkey.; 4 Department of Cardiovascular Surgery, Istanbul Mehmet Akif Ersoy Thoracic and Cardiovascular Surgery Training and Research Hospital, Istanbul, Turkey.

**Keywords:** Endocarditis, Thoracic Surgery, Mortality, Abscess, Alanine Transaminase, Hospital Mortality

## Abstract

**Introduction:**

Infective endocarditis is a disease that progresses with morbidity and mortality, afecting 3-10 out of 100,000 people per year. We conducted this study to review the early outcomes of surgical treatment of infective endocarditis.

**Methods:**

In this retrospective study, 122 patients who underwent cardiac surgery for infective endocarditis in our clinic between November 2009 and December 2020 were evaluated. Patients were divided into two groups according to in-hospital mortality. Demographic, echocardiographic, laboratory, operative, and postoperative data of the groups were compared.

**Results:**

Between November 3, 2009, and December 7, 2020, 122 patients were operated for infective endocarditis in our hospital. Emergency surgery was performed in nine (7.3%) patients. In-hospital mortality occurred in 23 (18.9%) patients, and 99 (81.1%) patients were discharged. In-hospital mortality was related with older age, presence of periannular abscess, New York Heart Association class 3 or 4 symptoms, low albumin level, high alanine aminotransferase level, and longer cross-clamping time (*P*<0.05 for all).

**Conclusion:**

The presence of paravalvular abscess was the most important prognostic factor in patients operated for infective endocarditis.

**Table:** 

Abbreviations, Acronyms & Symbols	
**ALT**	**= Alanine aminotransferase**
**AST**	**= Aspartate aminotransferase**
**BUN**	**= Blood urea nitrogen**
**CI**	**= Confidence interval**
**CRP**	**= C-reactive protein**
**HGB**	**= Haemoglobin**
**IABP**	**= Intra-aortic balloon pump**
**ICU**	**= Intensive care unit**
**IE**	**= Infective endocarditis**
**MRCNS**	**= Methicillin-resistant coagulase-negative staphylococci**
**MRSA**	**= Methicillin-resistant *Staphylococcus aureus***
**MSCNS**	**= Methicillin-sensitive coagulase-negative staphylococci**
**MSSA**	**= Methicillin-sensitive *Staphylococcus aureus***
**NYHA**	**= New York Heart Association**
**OR**	**= Odds ratio**
**P LT**	**= Platelet**
**SD**	**= Standard deviation**
**WBC**	**= White blood cell**

## INTRODUCTION

Infective endocarditis (IE) is a disease that progresses with morbidity and mortality, afecting 3-10 out of 100,000 people per year^[1]^. Despite early diagnosis and surgical interventions, hospital mortality was 17.1% in the European Infective Endocarditis (or EUROENDO) study published in 2019^[2]^. Surgical intervention in patients with IE is required because of heart failure, uncontrollable infection, and prevention of embolism. Almost half of IE patients undergo heart surgery during hospitalization^[3]^.

We conducted this study to review the early outcomes of surgical treatment of IE and to explain the impact of demographic, clinical, echocardiographic, and intraoperative parameters on in-hospital mortality and morbidity of IE patients after surgical treatment.

## METHODS

In this retrospective case-control study, patients who underwent cardiac surgery for IE in our clinic between November 2009 and December 2020 were evaluated. During this period, a total of 122 patients were operated for IE in our clinic. Their baseline demographic data, echocardiographic data, performance status, laboratory data, operative data, and postoperative status were comprehensively collected. Blood cultures were taken from all patients at the time of admission. Empirical broad-spectrum antibiotics were administered to patients with no known recent blood culture results. Then, specific treatment was arranged according to hemoculture and antibiogram results. Routine laboratory tests and blood, valves, and vegetations cultures were performed during hospitalization and treatment. Transesophageal echocardiography was performed after transthoracic echocardiography in all patients to determine surgical strategy. Surgical procedures for IE were performed using conventional cardiopulmonary bypass. All infected tissue was resected, and a physiological or anatomical surgical reconstruction was performed.

We defined emergency surgery as an operation with a refractory cardiac problem, which will not respond to any treatment other than cardiac surgery, and where there should be no delay in operative intervention. Hospital mortality was defined as mortality occurring within 30 days postoperatively or without discharge. This study was approved by the local ethics committee of our hospital (2018-23) and complies with the standards of the Declaration of Helsinki and current ethical guidelines.

### Statistical Analysis

Statistical analyses were per formed using the IBM Corp. Released 2015, IBM SPSS Statistics for Windows, version 23.0, Armonk, NY: IBM Corp. Continuous variables are expressed as mean ± standard deviation and categorical data as proportions throughout the manuscript. Categorical variables were compared using the χ2 test or Fisher’s exact test, and independent continuous variables were compared by the unpaired Student’s *t*-test or Kruskal-Wallis test as appropriate. Logistic regression analysis was performed to determine the predictors of in-hospital mortality. *P*-value < 0.05 was considered statistically significant.

## RESULTS

Between November 3, 2009, and December 7, 2020, 175 patients were hospitalized for IE in our clinic, and 122 (69.7%) patients were operated. Of the operated patients, 84 were male (68.9%) and their mean age was 52.53±15.10 years. There were seven (5.4) patients with a history of IE. Clinical features of the patients were as follows: New York Heart Association (NYHA) Functional Classification class 3 or 4 dyspnea (33.6%), fever exceeding 38°C (49.2%), and history of arterial embolism or stroke (17.2%). Emergency surgery was performed in nine (7.3%) patients. Coagulase-negative staphylococci were the most common pathogens causing IE in 25 patients — methicillin-resistant coagulase-negative staphylococci in 18 (14.75%), methicillin-sensitive coagulase-negative staphylococci in seven (5,74%) —, followed by streptococci in 11 (9.01%), *Staphylococcus aureus* in 10 (methicillin-sensitive *S. aureus* in seven [5.74%], methicillin-resistant *S. aureus* in three [2.46%]), *Enterococcus faecalis* in nine (7.38%), *Candida* in three (2.46%), *Escherichia coli* and *Stenotrophomonas* in two (1.64%), and *Brucella* in one (0.82%) patient. Blood cultures were negative in 73 patients (48.4%) (Table 1).

**Table 1:** Demographics and clinical characteristics of all infective endocarditis patients.

Patients’ Characteristics	n (%)/mean±SD
***Demographic feature***	
Age, years	52.53±15.10
Male, n (%)	84 (68.9)
History of infective endocarditis	7 (5.7)
***Preoperative clinical feature***	
NYHA class 3 or 4 symptoms	41 (33.6)
Body temperature > 38°C, n (%)	60 (49.2)
Previous emboli or stroke, n (%)	21 (17.2)
Emergency surgery, n (%)	9 (7.3)
Duration of antibiotic use, days	17.18±15.56
***Preoperative echocardiographic data***	
Right heart endocarditis	8 (6.6)
Ejection fraction	55.49±10.28
Pulmonary arterial pressure, mmHg	43.07±13.11
Vegetation > 1 cm, n (%)	49 (40.2)
Presence of periannular abscess, n (%)	16 (13.1)
***Vegetation site***	
Native aortic valve, n (%)	34 (27.9)
Native mitral valve, n (%)	32 (26.2)
Native tricuspid valve, n (%)	3 (2.5)
Multiple native valve, n (%)	13 (10.6)
Prosthetic aortic valve, n (%)	12 (9.8)
Prosthetic mitral valve, n (%)	20 (16.4)
Multiple prosthetic valve, n (%)	3 (2.5)
Device, n (%)	5 (4.1)
***Identified microorganism***	
Coagulase-negative staphylococci	
MRCNS	18 (14.75)
MSCNSA	7 (5.74)
*Staphylococcus aureus*	
MSSA	7 (5.74)
MRSA	3 (2.46)
*Streptococcus*	11 (9.01)
*Enterococcus faecalis*	9 (7.38)
*Candida*	3 (2.46)
*Stenotrophomonas*	2 (1.64)
*Escherichia coli*	2 (1.64)
*Brucella*	1 (0.82)
Negative blood culture	57 (46.7)
***Operational data***	
Mitral valve replacement, n (%)	46 (37.7)
Mitral valve repair, n (%)	12 (9.8)
Mitral valve repair and tricuspid annuloplasty, n (%)	6 (4.9)
Aortic valve replacement, n (%)	25 (20.5)
Aortic and mitral valve replacement, n (%)	19 (15.6)
Bentall procedure, n (%)	7 (5.7)
Right heart or device surgery, n (%)	7 (5.7)
Cardiopulmonary bypass time, min	152.76±84.31
Cross-clamping time, min	112.20±73.51

MRCNS=methicillin-resistant coagulase-negative staphylococci; MRSA=methicillin-resistant *Staphylococcus aureus*; MSCNS=methicillin-sensitive coagulase-negative staphylococci; MSSA=methicillin-sensitive *Staphylococcus aureus*; NYHA=New York Heart Association; SD=standard deviation

In-hospital mortality occurred in 23 (18.9%) patients, and 99 (81.1%) patients were discharged. Demographic, preoperative laboratory, and clinical characteristics of patients with and without in-hospital mortality were compared (Table 2). In-hospital mortality was related with older age, presence of periannular abscess, NYHA class 3 or 4 symptoms, low albumin level, high alanine aminotransferase (ALT) level, and longer cross-clamping time (*P*<0.05 for all).

**Table 2:** Comparison of demographic, preoperative laboratory, and clinical characteristics between patients with in-hospital mortality and patients without in-hospital mortality.

	Patients without in-hospital mortality (n=99)	Patients with in-hospital mortality (n=23)	*P*-value
Patients’ Characteristics	n (%)/mean±SD	n (%)/mean±SD	
***Demographic feature***			
Age, years	49.83±14.53	64.13±11.81	0.001*
Male, n (%)	69 (82.1)	15 (17.9)	0.676
History of infective endocarditis	6 (6.1)	1 (4.3)	0.750
***Preoperative clinical feature***			
NYHA class 3 or 4 symptoms	28 (28.3)	13 (56.5)	0.01*
Body temperature > 38°C, n (%)	47 (47.5)	13 (56.5)	0.434
Previous emboli or stroke, n (%)	14 (14.1)	7 (30.4)	0.062
Emergency surgery, n (%)	8 (8.1)	1 (4.3)	0,537
Duration of antibiotic use, days	17.49±16.05	15.82±3.45	0.645
***Preoperative echocardiographic data***			
Right heart endocarditis	8 (8.1)	0 (0)	0.158
Ejection fraction	57.49±16.05	55.82±13.45	0.645
Pulmonary arterial pressure, mmHg	43.12±13.32	42.87±12.42	0.937
Vegetation > 1 cm, n (%)	39 (39.4)	13 (56.5)	0.135
Presence of periannular abscess, n (%)	9 (9.1)	7 (30.4)	0.006*
***Preoperative laboratory value***			
WBC (109/L)	10.43±6.28	10.08±4.36	0.799
PLT (109/L)	278.31±130.96	253.13±104.02	0.391
HGB (g/dL)	10.83±3.7	9.61±1.72	0.127
Creatinine	1.25±1.28	1.47±1.11	0.443
BUN	21.85±12.23	26.91±16.88	0.101
Albumin (g/L)	37.84±8.56	31.78±7.91	0.020*
ALT (U/L)	24.87±40.42	48.78±75.44	0.036*
AST (U/L)	30.57±41.70	48.81±55.18	0.082
CRP	55.49±67.89	65.01±45.36	0.525
Procalcitonin	4.55±11.17	0.94±1.35	0.373
Sedimentation rate	62.84±33.65	81.5±40.71	0.132
***Operative data***			
Cardiopulmonary bypass time, min	146.63±82.70	179.13±87.94	0.096
Cross-clamping time, min	104.07±66.36	147.21±92.34	0.011*
Cardiac reoperation	37 (37.4)	11 (47.8)	0,355

*P<0.05 is considered as significant.

ALT=alanine aminotransferase; AST=aspartate aminotransferase; BUN=blood urea nitrogen; CRP=C-reactive protein; HGB=haemoglobin; NYHA=New York Heart Association; PLT=platelet; SD=standard deviation; WBC=white blood cell

Except for wound complication and focal neurological deficit, other complications were higher in the group of patients with in-hospital mortality. But there was no statistically significant diference between the two groups in terms of intensive care unit stay (Table 3). Postoperative mesenteric hemorrhage was seen in one patient. Tracheostomy was performed in one patient due to prolonged hospitalization. Nine patients were rehospitalized due to pleural or wound complications.

**Table 3:** Comparison of postoperative complications between patients with in-hospital mortality and those without in-hospital mortality.

	Patients without in- hospital mortality (n=99)	Patients with in-hospital mortality (n=23)	*P*-value
Low cardiac output syndrome, n (%)	4 (4)	14 (60.9)	< 0.0001*
Inotrope requirement, n (%)	24 (24.2)	22 (95.7)	< 0.0001*
Global neurological deficit, n (%)	7 (7.1)	13 (56.5)	< 0.0001*
Focal neurological deficit, n (%)	4 (4)	2 (8.7)	0.352
Arrhythmia, n (%)	21 (21.2)	14 (60.9)	< 0.0001*
Temporary pacemaker requirement, n (%)	7 (7.1)	5 (21.7)	0.033*
Lung parenchyma complications (atelectasis, pneumonia, etc.), n (%)	12 (12.1)	7 (30.4)	0.029*
Pleural complications, n (%)	17 (17.2)	12 (52.2)	< 0.0001*
Acute kidney injury, n (%)	15 (15.2)	14 (60.9)	< 0.0001*
Hemodialysis requirement, n (%)	4 (4)	12 (52.2)	< 0.0001*
Acute liver injury, n (%)	4 (4)	5 (21.7)	0.003*
Re-exploration for bleeding, n (%)	8 (8.1)	14 (60.9)	< 0.0001*
Wound complication, n (%)	10 (10.1)	3 (13)	0.680
Blood transfusion > 3 units, n (%)	23 (23.2)	15 (65.2)	< 0.0001*
IABP, n (%)	1 (1)	5 (21.7)	< 0.0001*
ICU stay, days	4.32±9.687	7.26±7.910	0.179

*P<0.05 is considered as significant. IABP=intra-aortic balloon pump; ICU=intensive care unit

Univariate and multivariate analyses were performed to identify independent risk factors related to in-hospital mortality. Univariate variables with *P*<0.05 were included in the multivariate analysis. Table 4 shows that older age, NYHA class 3-4 symptoms, and presence of periannular abscess were independently associated with in-hospital mortality after IE surgery in the multivariate analysis.

**Table 4:** Univariate and multivariate analyses for risk factors related to in-hospital mortality.

	Univariate analysis			Multivariate analysis		
	OR	95% CI	*P*-value	OR	95% CI	*P*-value
Age, years	1.093	1.043-1.146	< 0.000	1.074	1.024-1.127	0.003*
Previous emboli or stroke	2.656	0.927-7.612	0.069			
NYHA class 3 or 4 symptoms	3.296	1.296-8.382	0.012	3.152	1.006-9.873	0.049*
Body temperature > 38°C	1.438	0.577-3.587	0.436			
Presence of periannular abscess	4.375	1.425-13.433	0.010	4.823	1.066-21.816	0.041*
Ejection fraction	0.992	0.951-1.036	0.726			
Cross-clamping time	1.007	1.001-1.013	0.020	1.004	0.997–1.011	0.224
Cardiopulmonary bypass time	1.004	0.999-1.009	0.108			

*P<0.05 is considered as significant. CI=confidence interval; NYHA=New York Heart Association; OR=odds ratio

## DISCUSSION

The epidemiology of IE has changed significantly over the past three decades^[4]^. Mean age of the patients in our study was 52.53 years; compared to developed countries, patients with IE were mostly young^[5,6]^. The male rate was found to be 68.9%, which is consistent with male prevailing in the literature^[7]^. In our study, 7.3% of the patients were operated under emergency conditions. The duration of antibiotic use before the operation was 17.18 days in all patients.

In our cohort, 67.3% of the patients were operated for native valve endocarditis and 32.7% for prosthetic valve or cardiac device endocarditis. These data are similar to the International Collaboration on Endocarditis (or ICE) data published in 2009^[8]^. The most common pathogen in our study was staphylococci, similar to the literature^[9]^. The reason for the high culture negativity in our study may be the patients referred to our hospital. It can be explained by the negative blood culture taken from patients diagnosed with IE in another hospital and started on antibiotic therapy^[10]^.

In-hospital mortality rate was 18.9%, close to the two large international registries^[2,8]^.

As shown in our study, mortality was higher in elderly patients with IE. The high incidences of IE in the elderly and its clinical and echocardiographic features have been emphasized in many studies^[11,12,13]^.

No significant relationship was found between vegetation length > 1 cm and in-hospital mortality. However, there are many studies in the literature showing a relationship between vegetation size, in-hospital mortality, and embolic events^[14]^. In our study, we found a relationship between periannular abscess and in-hospital mortality. The presence of periannular abscess increased four times in-hospital mortality. Radical debridement of abscess cavities is an essential procedure in cardiac surgery and is important in active IE. In most cases, reconstruction using a pericardial patch is required to close the abscess cavity^[15]^. Necessary aggressive surgical treatment results in high complication and mortality rates. In the in-hospital mortality group, the serum albumin level was found to be low in the preoperative period. Hypoalbuminemia increases mortality as it is associated with malnutrition and frailty^[16]^.

### Limitations

The limitations of this study were the small number of patients, the review of short-term results, and the fact that it was a retrospective study. The study ofered the opportunity to evaluate surgically treated IE patients from a single referral tertiary care center.

## CONCLUSION

Although in-hospital mortality was related with older age, presence of periannular abscess, NYHA class 3 or 4 symptoms, low albumin level, high ALT level, and longer cross-clamping time in univariate analysis, multivariate analysis showed that the presence of paravalvular abscess was the most important prognostic factor in patients operated for IE.
